# Hand transplant– a challenge in immunological management of patients

**Published:** 2011-08-25

**Authors:** A Moise, I Constantinescu, B Serbanescu, CV Gingu, DG Zamfirescu, I Lascar

**Affiliations:** * ‘Carol Davila’ University of Medicine and Pharmacy, Immunology of Transplantation, Fundeni–Centre for Immunogenetics and Virology, Bucharest Romania; ** ‘Carol Davila’ University of Medicine and Pharmacy, Fundeni–Institute of Uronephrology and Renal Transplantation, Bucharest Romania; *** ‘Carol Davila’ University of Medicine and Pharmacy, Plastic Surgery and Reconstructive Microsurgery Clinic, Bucharest Emergency Hospital Romania

**Keywords:** composite tissue allotransplantation, immunology, tolerance

## Abstract

The concept of composite tissue allotransplantation (CTA) for restoration of congenital or acquired deformities is not new and the recent success of clinical composite tissue allotransplantation (CTA) attests to the fact that composite tissue allografts have tremendous potential in these life–enhancing reconstructions. A hand transplant, unlike a solid organ transplant, involves multiple tissues (skin, muscle, tendon, bone, cartilage, fat, nerves and blood vessels) and can be considered the ‘gold standard’ in CTA. In this regard, no other organ or tissue transplant matches the hand transplant in its immunogenicity as well as complexity. Development of assays that allow us to monitor the current state of an immune response (rejection/tolerance) is of great interest and  requires an in–depth understanding of the complex and rare phenomenon of tolerance.

## Review

Each year, an estimated 7–million people in the USA need composite tissue reconstruction because of surgical excision of tumors, accidents and congenital malformations. The recent success of clinical composite tissue allotransplantation (CTA) attests to the fact that composite tissue allografts have tremendous potential in these life–enhancing reconstructions.

The concept of CTA for restoration of congenital or acquired deformities is not new. In fact, one of the first accounts of transplantation dates back to c. 348 A.D. in which the sainted twins Cosmas and Damien replaced the gangrenous, cancerous leg of a sleeping man with that of a recently deceased Ethiopian Moor.  Then in the 16th century in Bologna, Italy, Gaspare Tagliacozzi, whom many regard as the ‘father of plastic surgery’, reportedly used a flap of tissue transplanted from a slave to reconstruct the severed nose of a man. In November 1997, in Louisville, Kentucky, USA was held the 1st International Symposium on CTA. At this meeting, reconstructive and transplant surgeons, immunologists, scientists and ethicists from around the world came together to discuss ‘the scientific, clinical, and ethical barriers standing in the way of performing the first successful human hand transplant’ [1] . Based on these findings, on September, 1998, was performed the first successful human hand transplant using tacrolimus/MMF/prednisone–based combination therapy, in Lyon, France by a team directed by J.–M. DUBERNARD. A 48–year old man with a right–hand amputation received a forearm transplant harvested from a 41–year old man in cadaveric (brain dead) status. The procedure was not considered by all as an advance in hand surgery [2], but it will be remembered as a major step in the history of Man, as were the first kidney (Murray, 1954) and heart transplantations (Barnard, 1967) [3]. 

The procedure is for individuals who have experienced the difficult loss of a hand or forearm due to: (1) trauma; (2) life saving interventions that caused permanent injury to the hand or forearm.  At this time, hand transplant procedure is not being considered for congenital anomalies, loss of a limb due to cancer or for leg amputations. Further research is needed in these areas. This procedure is not being considered for individuals whose injury is limited to fingers. The prospective patient should otherwise be healthy.

Tests required for further evaluation include the following, but are not limited to:

- X–rays
- Extensive blood work
- Physical measurements of the affected limb
- Psychiatric evaluation and psychological projective testing
- Consults with transplant surgeon, hand and microsurgeon, social worker, physical therapist, orthotist, primary care physician and other physician disciplines as appropriate
- Other tests as indicated such as gastrointestinal tests, etc.

Donated limbs would come from brain dead living donors similar to solid organ transplants. From a surgical point of view, harvesting tissues from a cadaveric donor gives several advantages which free the surgeon from the major constraints of traditional reconstruction. First, tissue allotransplantation obtains the preeminent objective of any tissue reconstruction; the ‘like with like’ replacement, … where a thumb would be reconstructed with a thumb but not with a toe and another major advantage of allografts is the avoidance of any donor site morbidity which liberates the surgeon from the dilemma of healthy tissue destruction, a drawback of any reconstruction by autologus tissues [3].

Composite tissue allotransplantation is not a new technique, but a new practice, that couples the rules of microsurgical reconstruction and the rules of human organ transplantation. The world experience in human hand transplantation to date includes 50 transplants performed in 36 recipients (www.handregistry.com). Overall the functional outcomes and patient satisfaction have been reported to be good [5,6]. Recovery of motor function enabled the patients to perform most daily activities, including eating, driving, grasping objects, riding a bicycle or a motorbike, shaving, using the telephone and writing [7]. 

A hand transplant, unlike a solid organ transplant, involves multiple tissues (skin, muscle, tendon, bone, cartilage, fat, nerves and blood vessels) and can be considered the ‘gold standard’ in CTA. Accordingly, no other organ or tissue transplant matches the hand transplant in its immunogenicity as well as complexity [4]. In that experience, the number of acute rejection episodes during the first year has been high when compared to more recent reports in organ transplantation. Reports indicate that the majority of patients demonstrated at least one episode of acute rejection in the first year, and that skin was the primary target of the immune response [8–18]. Repeated episodes were observed in some patients beyond the first year after transplantation [11–12]. The high frequency and severity of acute rejection in hand transplantation has been attributed to the high immunogenicity of the skin, which forms a major component of the graft. The high antigenicity of the skin can, in part, be related to the high proportion of potent antigen-presenting cells (Langerhans cells) and keratinocytes that express major histocompatibility complex (MHC) I constitutively, and MHC II, intercellular adhesion molecule 1 (ICAM)–I and proinflammatory cytokines upon stimulation [14,15]. Also, viral infections, in particular cytomegalovirus (CMV), have been postulated to trigger the episodes [16]. A major focus of current transplantation research is the development of strategies to obviate the need for immunosuppressive drugs by inducing specific tolerance to transplanted tissues. Strict definitions of transplantation tolerance include impaired responses to donor antigens with maintenance of immune responsiveness to third–party and non–donor antigens. Development of assays that allow us to monitor the current state of an immune response (rejection/tolerance) is of great interest and  requires an in–depth understanding of the complex and rare phenomenon of tolerance. The rarity of spontaneous transplantation tolerance and the difficulty of identifying such individuals pose major challenges in studying tolerance. In addition, the mechanisms of tolerance probably are numerous, may change over time, and vary depending on the organs involved. It now has become increasingly clear that tolerance in experimental rodent transplant models may differ from clinical transplantation in humans.

How Can We Measure Immunologic Tolerance in Humans? Immune monitoring assays that currently are in development can be divided broadly into two major categories: donor– antigen specific and antigen nonspecific [17].

### Antigen–Specific Assays

The development of immunologic memory and antigen specificity are hallmarks of the adaptive immune system. Therefore, assays that evaluate donor-specific responses of recipient lymphocytes are likely to be informative in transplantation. Traditional assays of  T cell reactivity that reflect antigen-specific responses include the mixed leukocyte reaction (MLR), the cytotoxic T lymphocyte (CTL) assay, the ELISA, and the limiting dilution assay. With the exception of the CTL assay, the results of these assays have not been shown consistently to be correlated with the development of tolerance or the ability to wean immunosuppression. The promising new T cell assays are:

ELISPOT Assay: is a hybrid that combines features of a MLR and an ELISA assay in that responder/recipient T cells are cultured with inactivated stimulator/donor or third–party cells in tissue culture plates that are coated with an antibody that is specific for the cytokine of interest (many cytokines have been studied, including IFN–gamma, IL–2, IL–4, IL–5, and IL–10). After a fairly brief culture period, the cells are washed away and the bound cytokine is detected, using labeled secondary antibodies and an automated plate reader. Each spot that is detected represents a cell that had been primed to the stimulating antigen(s) in vivo (effector or memory T cells). Thus, this assay measures the frequency of previously activated or memory T cells that respond to donor antigens by producing a selected cytokine rather than the total amount of cytokine that is produced and secreted into supernatant. Obtaining and storing sufficient numbers of donor cells to perform the assay repeatedly is a practical limitation, particularly in recipients of deceased–donor organs.

Transvivo delayed–type hypersensitivity (DTH) assay: cells that are isolated from patients after transplantation are injected into the footpads or ears of immunodeficient mice together with donor antigen. Recipient cells that respond to donor antigen produce a DTH reaction that is quantified by measuring the resultant swelling with a caliper. Like the ELISPOT assay, recipient T cells can be exposed to donor antigen in the form of whole, inactivated cells or donor proteins. A study of three functionally tolerant transplant recipients demonstrated that all three had intact DTH responses to third-party stimulator cells but absent DTH responses to donor antigens [18]. The transvivo DTH assay may be more useful for detecting patients with established tolerance than for making predictions about which patients who still are receiving immunosuppressive drugs may develop tolerance in the future.

Tetramer Staining: Tetramers consist of four MHC–peptide complexes that are linked covalently to a fluorochrome. Such multimeric peptide–MHC complexes can bind to the TCR of T cells that are specific for the peptide–MHC molecule complex [19]. The major potential of MHC tetramers is the direct visualization of antigen– specific T cells in vivo regardless of their function or ability to produce cytokines  and the possibility of monitoring peptide–specific T cells over time with very small volumes of blood. MHC tetramers were used recently to monitor minor–histocompatibility antigen–specific T cells in bone marrow recipients [20] and may have the potential for monitoring multiple autoimmune diseases [19].

Measurement of Cell Proliferation by carboxy–fluorescein diacetate succinimidyl ester (CFSE) Labeling: This assay measures the proliferative response of recipient lymphocytes that are cultured or stimulated with inactivated donor cells for a period of several days. Flow cytometry has been used to measure the dilution of the dye CFSE that segregates equally between daughter cells with each cell doubling. Despite the theoretic appeal of this assay, to date, no human studies have demonstrated a correlation between donor antigen–induced proliferation and the ability to wean immunosuppression or the development of tolerance.

Flow Cytometric Detection of Intracellular Cytokines: This method allows the individual characterization of a large number of cells. With multiparametric staining, it can demonstrate coexpression of different cytokines in individual cells. However, this assay involves specific activation procedures and use of inhibitors of intracellular transport, which can limit the viability of the cells. Another issue is the limited sensitivity of the assay.

### Non–Antigen–Specific Assays

Phenotyping of Recipient Immune Cells: Regulatory cells have been shown to be important for controlling immune responses in a number of pathogenic disease processes as well as after transplantation. Several types of regulatory cells have been identified on the basis of their phenotypes, including CD4^+^CD25^high^, CD3^+^CD4^-^CD8^-^, CD8^+^CD28^-^, and NK1.1^+^.  Although quantification and characterization of regulatory T cells has the potential to identify patients with predilection toward ‘tolerance’  no systematic studies have been performed to date.

Characterization of the TCR Repertoire: It has been hypothesized that the T cell component of the immune response to numerous self and foreign antigens is dominated by T cells using a limited number of TCR. This suggests a perturbation in the T cell repertoire that potentially could be measured. TcLandscape is one method for characterizing changes in the TCR repertoire [21]. Briefly, this assay uses quantitative PCR, gel electrophoresis and DNA sequencing to determine the proportion of T cells that use each of the V beta chains and to determine the CDR3 length distributions of each V beta gene product. Depending on the patient's clinical status, overrepresented TCR could indicate the expansion of alloreactive T cells that are capable of mediating allograft rejection or the expansion of protective regulatory T cells. One major issue with this method is lack of donor specificity. This may affect the interpretation of the results as other antigens such as viral infections are likely to influence the T cell repertoire.

T Cell Responses to Polyclonal, Non–Antigen–Specific Stimulation: This assay is performed by stimulating whole blood (i.e., lymphocytes in the presence of circulating levels of immunosuppressive drugs) with phytohemagglutinin for 12 to 15 h. CD4 T cells are isolated by magnetic bead selection. The extent of early CD4^+^ T cell activation is reflected by the synthesis and accumulation of intracellular ATP that is measured after cell lysis. This assay was designed to reflect the global or net state of immunosuppression and thereby facilitate decisions related to dosing immunosuppressive drugs after transplantation.

Assays to Quantify Gene Expression: The basic concept of DNA microarrays is as follows: mRNA is reversetranscribed into cDNA, labeled with a fluorescent dye, and hybridized to the array. After any unbound sample is washed away, the array is scanned. The fluorescence intensity at a specific spot represents an individual gene that correlates directly with the abundance of this gene in the sample. Unfortunately, genetic data sets that are obtained by this method usually are highly complex and require novel methods and software tools to handle the large volume of data generated.

Proteomics: Several proteins can be generated from a single gene, depending on how the genetic information is read (transcribed) and how the resultant protein is modified after translation (posttranslational modification) [22]. Analysis of mRNA expression alone therefore is insufficient to determine whether the proteins encoded are really synthesized. Thus, the proteomic approach can complement nicely the gene expression findings. Protein microarrays are capable of providing a highthroughput approach to quantify both the amount of protein present and the function of individual proteins.

In conclusion, continued success in clinical CTA over the next few years could convince the transplant community to shed its skepticism, that all attempts at CTA are ambitious and misguided. The CTA area is among the newest of transplant areas. The immunology of CTA grafts is complex, making CTA tolerance more difficult to achieve than organ tolerance. It needs to be emphasized that any episodes of acute rejection should be prevented for perfect restoration of function as well as to minimize the risk of chronic rejection in CTA. Efficacious, safe and ethical clinical tolerance protocols could improve patient acceptance of CTA by providing an alternative to chronic immunosuppression. The safe and successful clinical application of tolerance–inducing strategies in patients after transplantation will depend on identifying assays that can detect and even predict the development or loss of tolerance.
